# Multiwalled carbon nanotubes enter broccoli cells enhancing growth and water uptake of plants exposed to salinity

**DOI:** 10.1186/s12951-016-0199-4

**Published:** 2016-06-08

**Authors:** Mª Carmen Martínez-Ballesta, Lavinia Zapata, Najla Chalbi, Micaela Carvajal

**Affiliations:** Plant Nutrition Department, Centro de Edafología y Biología Aplicada del Segura (CEBAS-CSIC), Campus Universitario de Espinardo, Edificio 25, 30100 Murcia, Spain; Laboratory of Extremophile Plants, Center of Biotechnology of Borj-Cedria (LEP-CBBC), P. O. Box 901, 2050 Hammam-Lif, Tunisia

**Keywords:** Aquaporin, *Brassica oleracea*, Multiwalled carbon nanotubes, Lipid composition, Root hydraulic conductance, Stomatal conductance

## Abstract

**Background:**

Carbon nanotubes have been shown to improve the germination and growth of some plant species, extending the applicability of the emerging nano-biotechnology field to crop science.

**Results:**

In this work, exploitation of commercial multiwalled carbon nanotubes (MWCNTs) in control and 100 mM NaCl-treated broccoli was performed. Transmission electron microscopy demonstrated that MWCNTs can enter the cells in adult plants with higher accumulation under salt stress. Positive effect of MWCNTs on growth in NaCl-treated plants was consequence of increased water uptake, promoted by more-favourable energetic forces driving this process, and enhanced net assimilation of CO_2_. MWCNTs induced changes in the lipid composition, rigidity and permeability of the root plasma membranes relative to salt-stressed plants. Also, enhanced aquaporin transduction occurred, which improved water uptake and transport, alleviating the negative effects of salt stress.

**Conclusion:**

Our work provides new evidences about the effect of MWCNTs on plasma membrane properties of the plant cell. The positive response to MWCNTs in broccoli plants opens novel perspectives for their technological uses in new agricultural practices, especially when 1plants are exposed to saline environments.

**Electronic supplementary material:**

The online version of this article (doi:10.1186/s12951-016-0199-4) contains supplementary material, which is available to authorized users.

## Background

The positive effects of engineered nanomaterials on plants comprised increased germination percentage and rate; length of root and shoot, and vegetative biomass of seedlings with the involvement of distinct physiological processes related to the plant water balance, photosynthetic activity and nitrogen metabolism. Thus, the water status of many crops was affected by different nano-materials. It has been observed that coated seeds with nano-membranes may regulate the capacity of water imbibition when the seeds are ready to germination. Also, magnetic particles in seeds allowed the detection of moisture content during seed storage [1]. Ag nanoparticles reduced the effects of microbial infection on gerbera flowers, increasing the water uptake and turgidity [2]. TiO_2_ nanoparticles enhanced nitrate uptake in Soybean (*Glycine max*) plants, increasing the ability to absorb/use water, and promoting the antioxidant system. Also, TiO_2_ regulated water absorption in spinach seeds, with an increased germination rate [3]. However, contrast results were found for the effect of engineered nanomaterials on plant water uptake and growth. Excess of metals nanoparticles resulted in toxic effects that involved a decrease of growth and abnormalities in cell division [4]. Copper/copper oxide (Cu/Cu_2_O) nanoparticles may block water channels reducing water uptake [5].

Since their discovery in 1991 [6], carbon nanotubes (CNTs) have proven to be the most interesting of the nano-materials due to their unique mechanical, electrical, thermal and chemical properties [7]. Their extraordinary physical properties give them potential uses in a multitude of fields; one that promises significant advances is biomedicine. In this area, they have been investigated for gene and drug delivery, diagnosis, tissue regeneration and implants. Therefore, most studies of CNTs have been related to tissues of humans and other animals [8], but investigation of their effects in plants is very scarce. Thus, the plant-CNTs interaction needs to be thoroughly investigated, from the molecular to the cellular and organ levels, integrating plant physiology to understand its complexity.

The first evidence of positive effects of multiwalled carbon nanotubes (MWCNTs) on crop plants was reported by Khodakovskaya et al. [9]. Using a range of concentrations from 10 to 40 mg l^−1^, they observed that CNTs could penetrate the coats of tomato seeds, increasing germination rates and stimulating growth in young seedlings. In a more-recent study, Khodakovskaya et al. [10] demonstrated that MWCNTs, introduced into the soil through watering, can affect the phenotype of adult tomato plants. These plants produced a similar quantity of leaves but two-time more flowers and fruits than plants grown in regular soil. This observation opens new perspectives for the use of CNTs as growth regulators in conventional agricultural systems. Also, in *Brassica juncea*, the application of suitable CNTs concentration increased the heights of the roots [11], germination rate and growth [12].

However, some reports have indicated negative effects of CNTs on plants [13]; At high concentrations, the accumulation of graphene decreased the root growth of red spinach and cabbage [14] as consequence of massive electrolyte leakage, indicating an oxidation stress mechanism and damage in the plasma membrane that may affect water and solute transport [15]. Fullerene was accumulated in the leaves following the symplastic route [16, 17] and the large aggregates accumulation within the vascular tissues may impede the appropriated translocation of water and nutrients to the aerial part [18]. Tiwari et al. have shown that maize seedlings grown in agar reduced dramatically their growth and water uptake after treatment with different concentrations of pristine MWCNTs [19].

The effect of CNTs was dependent on the size, concentration and solubility of the applied CNTs. Hence, there is a need for further investigation before the application of CNTs can be considered as a widely-applicable means of increasing crop growth and productivity [20]. In the study of the molecular mechanisms by which CNTs may act on plant physiology, a correlation between the activation of plant cell growth after MWCNTs exposure and the up-regulation of genes involved in cell division/cell wall formation and water transport, such as those controlling synthesis of aquaporins, was found [21]. There are many reports indicating that aquaporins are crucial for root water uptake, seed germination, cell elongation, reproduction and photosynthesis [22]. Also, the expression of a large number of aquaporin proteins has been shown to occur predominantly in roots and different experimental procedures have demonstrated, in various species, that aquaporins activity is linked to plant hydraulics during abiotic stress [23]. However, it has been also demonstrated that MWCNTs may create new pores in the cell wall and plasma membrane allowing water transport to develop tomato and wheat seedlings [16, 17].

As it has been found that several genes involved in the general plant response to stress were up-regulated after carbon nanohorns treatment [24], conferring a positive response, the effect of CNTs in plants under stress deserves further attention.

The inhibition of plant growth by salinity via its effects at the physiological, biochemical and molecular levels has been studied widely [25, 26]. The homeostasis of ions and water is the main physiological process that plants need to optimise in order to maintain growth in saline environments [27, 28]. Osmotic adjustment may help plant cells to withstand salt stress, specifically to cope with the water deficit generated by the salt-induced osmotic imbalance, through the maintenance of the water potential gradient between the plant and the substrate, which is necessary to conserve water uptake and cell turgor for growth [29, 30]. Therefore, salinity is probably the most-suitable abiotic challenge under which to investigate the effect of CNTs, due to the similarity of the processes involved to those of other environmental stresses.

Membrane transport proteins and the surrounding lipids are crucial regulators of nutrient and water acquisition and of the exchanges of molecules between subcellular compartments; hence, they are vital to the maintenance of turgor under saline conditions [31]. The trans-membrane water and solute fluxes are controlled by the abundance and activity of plasma membrane proteins [32]. Also, the early response of proteins to salinity may be involved in the modulation of growth-regulating mechanisms and membrane stability [33]. Also, changes in the chemical composition and physical properties of plasma membrane lipids have been observed as an adaptation to external salinity [34]. A decrease in the unsaturation of fatty acids has been reported, together with an increase of sitosterol [35]. In this sense, an increment in protein abundance in relation to the content of lipids in the plasma membrane was reported to play a role in the plasma membrane stability [36, 37]. In order to initiate further investigations at the intracellular level, the effect of MWCNTs on the plasma membrane should be elucidated. Therefore, in this work, we describe the results of applying commercial MWCNTs to control and salt-stressed (NaCl) broccoli plants in order to evaluate the effect of these synthetic CNTs on water and nutrient transport through the plasma membrane. The root hydraulic conductance, stomatal conductance, leaf water potential and CO_2_ fixation were measured as well as the mineral nutrient content, and the changes in the plasma membrane lipid composition were analysed and evaluated in relation to aquaporin abundance.

## Methods

### Plant material and growth conditions

Seeds of broccoli (*Brassicaoleracea* L. var. Italica) were pre-hydrated with de-ionised water and aerated continuously for 12 h. After this, the seeds were germinated in vermiculite, in the dark at 28 °C, for 2 days. They were then transferred to a controlled-environmental chamber, with a 16-h light and 8-h dark cycle with temperatures of 25 and 20 °C and relative humidities of 60 and 80 %, respectively. Photosynthetically-active radiation (PAR) of 400 µmol m^−2^ s^−1^ was provided by a combination of fluorescent tubes (Philips TLD 36W/83, Jena, Germany and Sylvania F36W/GRO, Manchester, NH, USA) and metal halide lamps (Osram HQI, T 400W, Berlin, Germany). After 3 days, the seedlings were placed in 15 l containers with continuously-aerated Hoagland nutrient solution [38]. After 1 week of growth, different treatments were applied for 7 days.

**Fig. 1:**
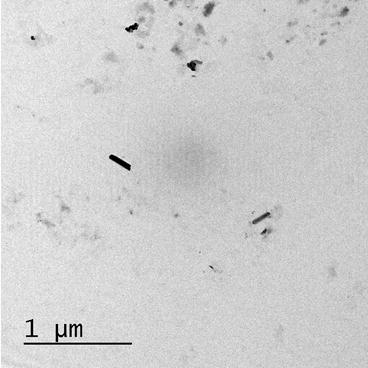
TEM image of an individual multi walled carbon nanotube (MWNTs). Inset *scale bar* 100 µm

The first experimental set consisted of broccoli seedlings grown in Hoagland nutrient solution with different concentrations of MWCNTs. This approach defined the basic response of the given plants to the MWCNT unambiguously, a baseline study that is necessary for each cultivar. The MWCNTs were purchased from Sigma-Aldrich (St. Louis, MO, USA), an established chemicals company and possessed reliable, quality-controlled specifications; purity [95 %, OD 6–9 nm, L 5 lm], approximately 5–20 graphitic layers, a core surrounded by a fused carbon shell, the remainder being multi-layer polygonal carbon nanoparticles and amorphous and graphitic carbon nanoparticles. Their size was 0.1–0.5 μm length (Fig. 1). MWCNTs were sonicated in ultrasound bath (Selecta S.L., Barcelona, Spain) at sonicator output power of 150 W during 40 min. These conditions determined an average size particle of 0.1–0.5 μm length.

The Hoagland solution was used as the control. The MWCNTs were added to the control medium at concentrations of 10, 20, 40 and 60 mg l^−1^: Another set of plants were treated with concentrations of 80, 100 and 120 mM NaCl. Six plants per treatment were chosen for fresh weight (FW) determination. Plant FW was directly recorded with a portable balance (Sartorius CP224S). For further studies the combination of 100 mM NaCl + 10 mg l^−1^ MWCNT was chosen.

The second experimental set consisted of broccoli seedlings grown in Hoagland solution with NaCl (100 mM), with or without the simultaneous presence of 10 mg l^−1^ MWCNT. Six plants per treatment were chosen for FW determination. The total dry weight (DW) was measured after after drying the plant in an oven at 70 °C until constant weight.

### Analysis of mineral elements

The concentrations of macronutrients (Ca, K, Mg, Na, P and S) were analysed in oven-dried samples of plant material (root and the third leaf), which had been ground finely in a mill grinder IKA model A10 (Staufen, Germany) to give particle sizes of 0.5–0.7 mm. The samples were digested in a microwave oven (CEM Mars Xpress, Mattheus, NC, USA) by HNO_3_–HClO_4_ (2:1) acid digestion. The elemental analysis was carried out using a Perkin–Elmer (Waltham, MA) 5500 model ICP emission spectrophotometer (Iris Intrepid II, Thermo Electron Corporation, Franklin, USA), at 589 nm and expressed as mg Kg^−1^ DW.

### Leaf gas exchange parameters

Stomatal conductance (G_s_) and net assimilation of CO_2_ (ACO_2_) were measured using a portable photosynthesis system (model LCA-4, ADC Bioscientific Ltd., Hoddesdon, UK) and a PLC-4N leaf chamber (11.35 cm^2^), configured to an open system. The third fully-expanded leaf was chosen for the analyses after the different treatments. Leaves were first equilibrated at a photon density flux of 400 μmol m^−2^ s^−1^ for at least 20 min. G_s_ and ACO_2_ were determined during the necessary seconds to stabilise the reading, once stabilised measurements were reordered every 10 s during 1 min. The measurements were made in the middle of the photoperiod in order to obtain the highest values. During all the measurements the cuvette temperature was 25 ± 2 °C, the CO_2_ concentration of the cuvette was 350 ± 5 ul l^−1^ and the leaf-to-air vapour pressure difference was 2.2 ± 0.3 kPa within the cuvette.

### Root hydraulic conductance (L_0_)

Root hydraulic conductance (L_0_) was measured by pressurising the roots using a Schölander chamber. The aerial parts of the plants were removed, and the base of the stem was sealed with silicone grease into a tapered glass tube. The plant was then placed in the pressure chamber, with the same nutrient solution in which it had been grown, and a gradual increase of pressure (from 0.1 to 0.5 MPa) was applied to the roots, thereby generating a range of sap flows that represented whole-plant transpiration rates. Sap was collected in Eppendorf tubes and weighed on a precision balance. The sap flow, J_V_, was expressed in mg (g root FW)^−1^ h^−1^ and plotted against pressure (MPa), the slope being the L_0_ value [mg g (root FW)^−1^ h^−1^ MPa ^−1^]. The measurements were made in the middle of the photoperiod, 1 week after applying the different treatments.

### Treatment with sodium azide

Classically, sodium azide has been applied as an aquaporin functionality inhibitor [39]; its effect may be due to the fact that it inhibits protein phosphorylation and causes acidification of the cytoplasm, both effects contributing to the closure of aquaporin channels [40]. Plants from each treatment were treated with sodium azide at a final concentration of 7 mM [41]. Whole plants were immersed in their respective treatments and sodium azide (7 mM), so that the root system was kept undisturbed during the treatment. The plants were maintained under submersion for 60 min to allow uptake by the roots. Plants not treated with sodium azide were kept as a control.

### Plasma membrane purification

Root plasma membranes were purified using the two-phase aqueous polymer technique described in Muries et al. [42]. Fresh roots (20 g) were chopped into approximately 1 mm^3^ cubes and vacuum-infiltrated with 40 ml of a buffer containing 500 mM sucrose, 10 % glycerol, 20 mM Na_2_EDTA, 20 mM EGTA, 50 mM NaF, 5 mM glycerophosphate, 1 mM 1,10-phenantroline, 1 mM Na_3_VO_4_, 0.6 % PVP, 5 mM ascorbic acid, 5 mM DTT and 0.5 mg l^−1^ leupeptin in 50 mM Tris-MES, pH 8.0. After buffer infiltration, the roots were homogenised using a pestle and mortar and filtered through a nylon cloth (240 μm pores). The filtrate was centrifuged at 10,000*g* for 15 min. The supernatant was recovered and centrifuged at 55,000*g* for 35 min, yielding a microsomal pellet which was resuspended in 0.33 M sucrose, 2 mM DTT, 10 mM NaF and 5 mM phosphate buffer (pH 7.8). Plasma membranes were purified from microsomes by partitioning in a two-phase system mixture with a final composition of PEG-3350 (Sigma)/Dextran-T500 (GE Healthcare), 6.3 % (w/w) each in the presence of 5 mM KCl, 330 mM sucrose, 2.5 mM NaF and 5 mM potassium phosphate (pH 7.8) The two-phase system was centrifuged for 5 min at 4000*g*. The resulting upper phase, enriched in plasma membranes, was washed in 9 mM KCl, 300 mM sucrose, 0.2 M EDTA, 0.2 M EGTA, 0.5 M NaF and 10 mM Tris–borate, pH 8.3, and centrifuged at 55,000*g* for 35 min; the resulting lower phase was resuspended in 1 ml of 9 mM KCl, 300 mM sucrose, 0.2 M EDTA, 0.2 M EGTA, 0.5 M NaF, 2 mg l^−1^ leupeptin, 1 M DTT and 10 mM Tris–borate, pH 8.3.

Lipid analysis was carried out in the plasma membrane of theses purified vesicles whereas an enrichment in hydrophobic proteins was performed [43], previous the immunoblotting assay.

The protein concentration of the plasma membrane intrinsic protein (PIP)-enriched fraction was determined with the RC DC Protein Assay kit (BioRad), using BSA as standard. Plasma membrane samples were stored at −80 °C before use.

### Gel electrophoresis and immunoblotting assay

Plasma membrane from the root tissues of broccoli plants was isolated as described previously. Protein (10 μg per lane) was loaded for 12 % sodium dodecyl sulphate–polyacrylamide gel electrophoresis (SDS–PAGE). The proteins had been denatured previously, by incubation at 56 °C for 20 min in the presence of 2 % (w/v) SDS and 100 mM DTT.

The proteins were transferred to a PVDF membrane and run for 20 min at 15 V, in an electrophoretic transfer cell (Trans-Blot SD cell, BioRad), using Towbin transfer buffer [44] with the addition of 0.05 % SDS. After this, the membrane was blocked for 1 h at room temperature, in tris-buffered saline (TBS) containing 2 % (w/v) skimmed dry milk. Then, the membrane was incubated for 1 h at room temperature in a buffer containing TBS with 0.05 % Tween 20, in the presence of an antibody (dilution 1:3000) raised against the first 42 N-terminus residues of *Arabidopsis* PIP1;1 (kindly provided by Prof. Dr. Anthony Schäffner) and another raised against a 17-amino-acid C-terminal peptide of Arabidopsis PIP2;2 (dilution 1:2000) (kindly provided by Dr. Veronique Santoni), shaking overnight at 4 °C. Goat anti-rabbit Immunoglobulin G (IgG) coupled to horseradish peroxidase was used as the secondary antibody (dilution 1:20,000). A chemiluminescent signal was developed using the West-Pico, Super Signal substrate (Pierce, Rockford, IL). The immunoblots were performed on samples from three independent experiments.

The intensity of each band was determined by a GS-800™ calibrated densitometer (Bio Rad, Barcelona, Spain). Background correction was performed by sampling membrane regions without spots and averaging their signals. The intensity of each band was estimated as the difference between the raw intensity of the protein of interest and the background. The scanned bands were normalised by calculating the ratio relative to the corresponding Coomassie-stained bands, thus ensuring that different intensities were not due to differences in the amount of protein loaded.

### Leaf water potential (Ψ_ω_)

The leaf water potential (Ψ_ω_) of the third fully-expanded leaf was measured in the middle of the photoperiod using the pressure chamber technique [45].

### Root plasma membrane lipid analysis

Sterol and fatty acids were determined as described by Mas et al. [46] and adapted by López-Perez et al. [31] in the root plasma membrane of broccoli plants. In an Eppendorf tube, a mixture of chloroform–methanol (1:2, 0.75 ml) was added to the membranes obtained (0.5 ml), along with b-cholestanol (20 μl, 0.1 mg ml^−1^)—used here as an internal standard for sterol analysis. Chloroform (CHCl_3_; 0.25 ml) was added and the mixture was shaken and centrifuged at 10,000*g* for 6 min. The CHCl_3_ layer was retained, evaporated to dryness under N_2_ (weighing this portion shows the amount of total lipids) and made up to 100 μl with CHCl_3_. For sterol analysis, 20 μl of the CHCl_3_ extract were placed in a glass vial (2 ml), evaporated to dryness under N_2_ and acetylated using pyridine (50 μl) and Ac_2_O (100 μl). After 2 h, the solvents were evaporated under N_2_, ethyl acetate (20 μl) was added and the sterol was analysed by gas chromatography (GC) using an HP5-bonded capillary column (30 m × 0.25 mm × 0.25 μm) coupled to a flame ionisation detector (FID), with H_2_ as carrier (1 ml min^−1^) and a temperature programme of 120–260 °C at 5 °C min^−1^, then 260–280 °C at 2 °C min^−1^ and finally 280–300 °C at 6 °C min^−1^. The injector and detector temperatures were 150 and 320 °C, respectively. Bound fatty acids were determined in 20–25 μl portions of the CHCl_3_ extract, by evaporating them to dryness under N_2_, transmethylating with sodium methoxide (0.5 N) in methanol (0.5 ml) and heating at 30 °C for 7 min. The resultant fatty acid methyl esters were extracted with hexane (1 ml), evaporated under N_2_, dissolved in ethyl acetate (20 μl) and analysed by GC using an HP5-bonded capillary column (30 m × 0.25 mm × 0.25 μm), with FID, He as carrier (1 ml min^−1^) and a temperature programme of 150–195 °C at 3 °C min^−1^, then 195–220 °C at 2 °C min^−1^ and finally 220–300 °C at 6 °C min^−1^. The injector and detector temperatures were 280 and 300 °C, respectively.

### Lipid/protein ratio in the plasma membrane

The total lipids were determined by weighing the lipid extraction phase for lipid analysis, after evaporation to dryness under N_2_. The protein concentration of the plasma-membrane-enriched fraction was determined as described before.

### Transmission electron microscopy

Root (8–10 cm from the tip), stem (3 cm from the base) and leaf (third leaf) samples were collected and then cut into small pieces (1–2 mm) and immersed in 2.5 % (v/v) glutaraldehyde and 3 % paraformaldehyde in 0.1 m sodium phosphate buffer (pH 7.2) for 2.5 h. After three 15 min washes with the buffer, the samples were postfixed in 1 % osmium tetroxide, in the same buffer, for 2 h. After this, three washes with phosphate buffer were performed. All fixed tissues were dehydrated in a graded series of ethanol (35, 50, 70, 96 and 100 %), then infiltrated, first with a propylene oxide and then with propylene oxide and Spurr’s resin mixture. The samples were then immersed in Spurr’s resin overnight at 4 °C. Finally, the samples were embedded in Spurr resin. Blocks were sectioned on a Leica EM UC6 ultramicrotome, collected on Formvar-coated copper grids and stained with uranyl acetate followed by lead citrate. Sections were examined using a Philips Tecnai 12 transmission electron microscope. For each treatment, an average of six samples from four different tissues was investigated. For each sample, 5–10 ultrathin sections were examined.

### Data analysis

Statistical analyses were performed using SPSS Release 18 for Windows. Significant differences between the values of all parameters were determined at *P* ≤ 0.05, according to Tukey’s test.

## Results

### Fresh weight

The fresh weight (FW) of the whole plants was determined after 7 days of growth at different concentrations of the MWCNTs (10, 20, 40, 60 mg l^−1^) (Additional file 1). There was a FW decrease at 20, 40 and 60 mg l^−1^ MWCNT, compared to the control value, while 10 mg l^−1^ MWCNTs produced a significant increase (32.75 %).

### Effect of MWCNTs on fresh weight under salt stress

**Fig. 2:**
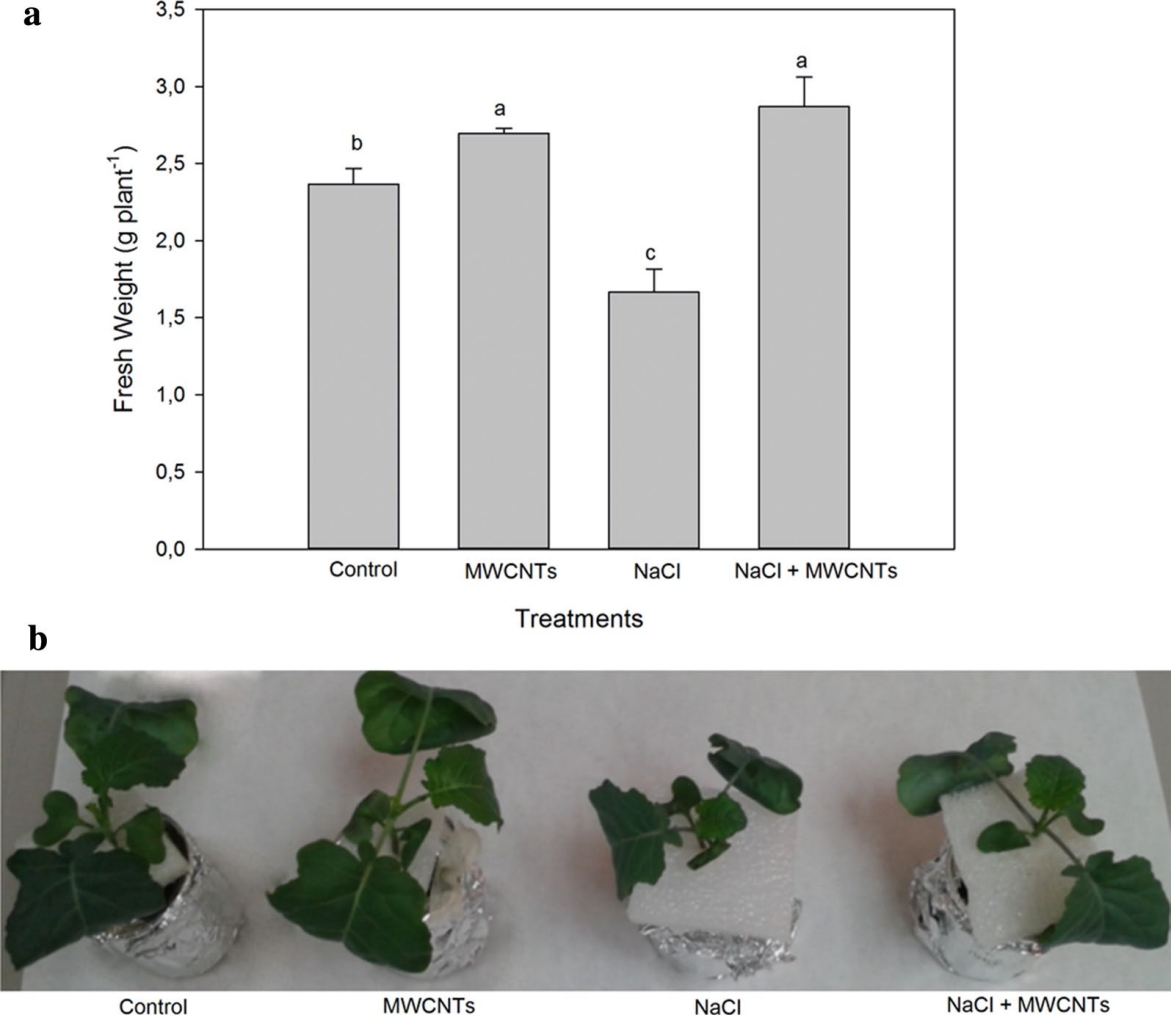
**a** Fresh weight (FW) (g plant^−1^) of control broccoli plants and plants treated with 10 mg l^−1^ MWCNTs, 100 mM NaCl and 100 mM NaCl + 0^.^10 mg l^−1^ MWCNTs. Mean values ± standard errors are shown (n = 6). *Bars* with different letters are significantly different (*p* < 0.05) according to the Tukey test. **b** Picture showing the effect of MWCNTs (10 mg l^−1^) on the growth of control and 100 mM NaCl-treated broccoli plants

The FWs were measured after application of salinity (100 mM NaCl) and 10 mg l^−1^ MWCNTs. The suitable concentration of NaCl was determined according to preliminary experiments where NaCl concentrations of 80, 100 and 120 mM were assayed (Additional file 2). The response of broccoli plants to salt stress has been well characterised in adult plants, but such young plants (14 days old) have not been used in previous experiments. Although a similar FW decrease was induced by both NaCl levels, 100 and 120 mM, we considered the latter inappropriate for broccoli plants [47]. Therefore, when 100 mM NaCl was supplied in combination with 10 mg l^−1^ MWCNTs, a positive effect on growth was observed, compared with the application of NaCl alone, with only a slight difference in FW with respect to control and MWCNTs-treated plants (Fig. 2). Similar results were obtained for DWs (Additional file 3) since the relative water content was similar (~92 %) in all treatments.

### Ion concentrations

The macronutrients analysis of the roots and shoots of broccoli plants grown under different treatments is shown in Table 1. In the roots, salinity reduced Ca, K, P and Mg independently of the MWCNTs addition. In the shoots, Ca and K levels were decreased by salt stress but, there were no significant differences in P, S and Mg content between treatments. The effect of MWCNTs on ion levels was observed in the roots and shoots of NaCl + MWCNTs treated-plants relative to the only NaCl addition, where higher Na accumulations were observed. In addition, in the shoots, a restoration of K levels by MWCNTs was found in saline-treated plants.

**Table 1:** Ca, K, Na, P, S and Mg (10^−4^ mg kg^−1^DW) levels in the root and shoot of control broccoli plants and plants treated with 10 mg l^−1^ MWCNTs, 100 mM NaCl and 100 mM NaCl + 0^.^10 mg l^−1^ MWCNTs

	Ca	K	Na	P	S	Mg
Root						
Control	2.30 ± 0.10 a	4.61 ± 0.30 a	0.12 ± 0.03 c	1.88 ± 0.10 a	0.65 ± 0.07 a	0.95 ± 0.07 a
MWCNT	2.07 ± 0.11 a	4.75 ± 0.51 a	0.11 ± 0.02 c	1.66 ± 0.12 a	0.71 ± 0.07 a	0.83 ± 0.07 a
NaCl	0.99 ± 0.04 b	3.46 ± 0.42 b	2.02 ± 0.18 b	1.05 ± 0.10 b	0.58 ± 0.06 a	0.62 ± 0.05 b
NaCl + MWCNT	0.70 ± 0.03 b	3.25 ± 0.29 b	2.34 ± 0.10 a	0.82 ± 0.08 b	0.63 ± 0.05 a	0.69 ± 0.04 b
Shoot						
Control	2.10 ± 0.11 a	4.57 ± 0.43 a	0.13 ± 0.05 c	0.54 ± 0.05 a	0.71 ± 0.06 ab	0.38 ± 0.04 a
MWCNT	2.48 ± 0.18 a	4.51 ± 0.27 a	0.09 ± 0.02 c	0.54 ± 0.03 a	0.96 ± 0.07 a	0.43 ± 0.03 a
NaCl	1.27 ± 0.17 b	2.36 ± 0.10 c	2.26 ± 0.28 b	0.46 ± 0.07 a	0.61 ± 0.06 b	0.33 ± 0.03 a
NaCl + MWCNT	1.34 ± 0.10 b	3.27 ± 0.12 b	3.11 ± 0.12 a	0.55 ± 0.05 a	0.59 ± 0.04 b	0.30 ± 0.03 a

Mean values ± standard errors are shown (n = 6). Bars with different letters are significantly different (*P* < 0.05) according to the Tukey test

### Stomatal conductance and net assimilation of CO_2_

**Fig. 3:**
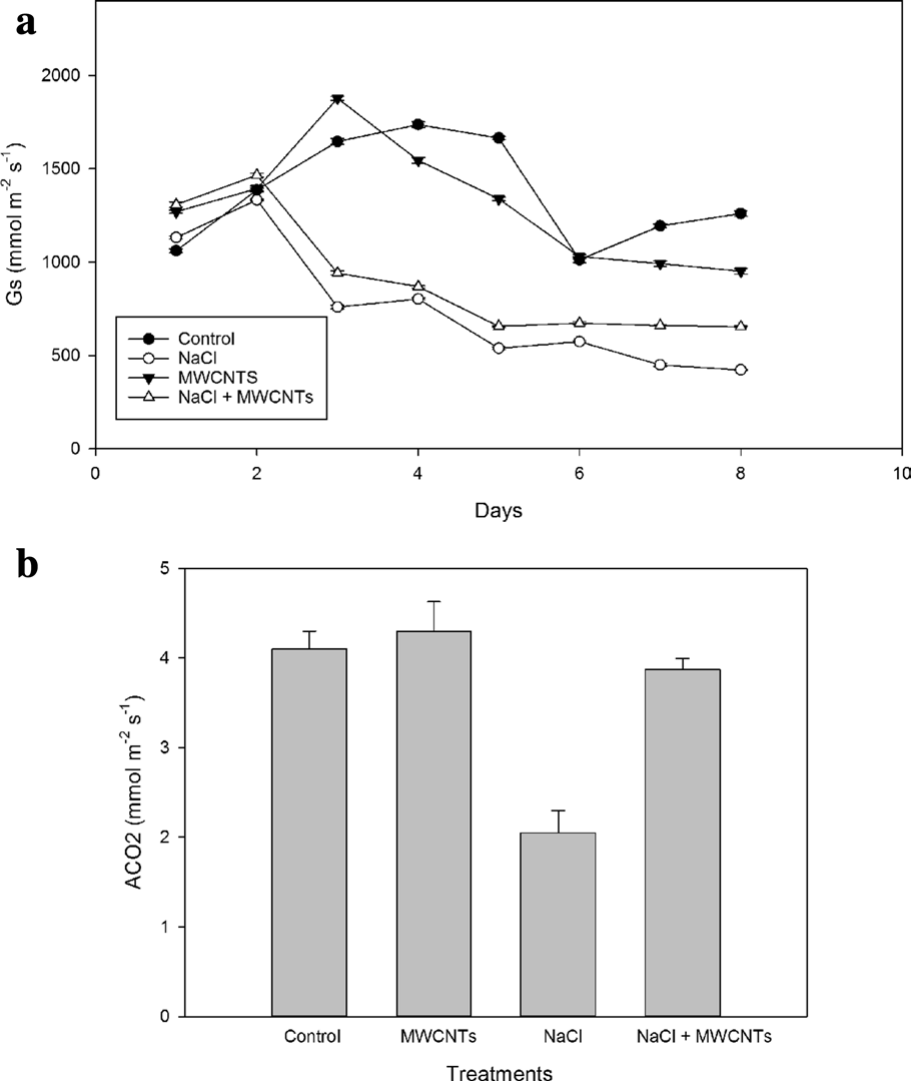
**a** Time-course of stomatal conductance (G_s_) (mmol m^−2^ s^−1^) and **b** Net assimilation of CO_2_ (ACO_2_) (mmol m^−2^ s^−1^) in the leaves of broccoli plants and plants treated with 10 mg l^−1^ MWCNTs, 100 mM NaCl and 100 mM NaCl + 0^.^10 mg l^−1^ MWCNTs. Mean values ± standard errors are shown (n = 6). *Bars* with *different letters* are significantly different (*P* < 0.05) according to the Tukey test

The stomatal conductance (G_s_) was measured each day after MWCNTs and NaCl treatment (Fig. 3a). The changes were observed after the first day of treatment. After 2 days, the G_s_ values decreased progressively in plants treated with NaCl (NaCl and NaCl + MWCNTs, but the decrease was higher for the plants treated only with NaCl. In control and MWCNTs-treated plants, G_s_ increased from the 2nd to the 4th day, but returned to the initial values after the 6th day.

Net assimilation of CO_2_ (ACO_2_) was measured on the same days as G_s_, but only the final values are shown since they are representative of every day (Fig. 3b). Only plants treated with NaCl showed significant decreases of ACO_2_. The other treatments did not affect ACO_2_—which remained unchanged from the first day of measurement.

### Root hydraulic conductance

**Fig. 4:**
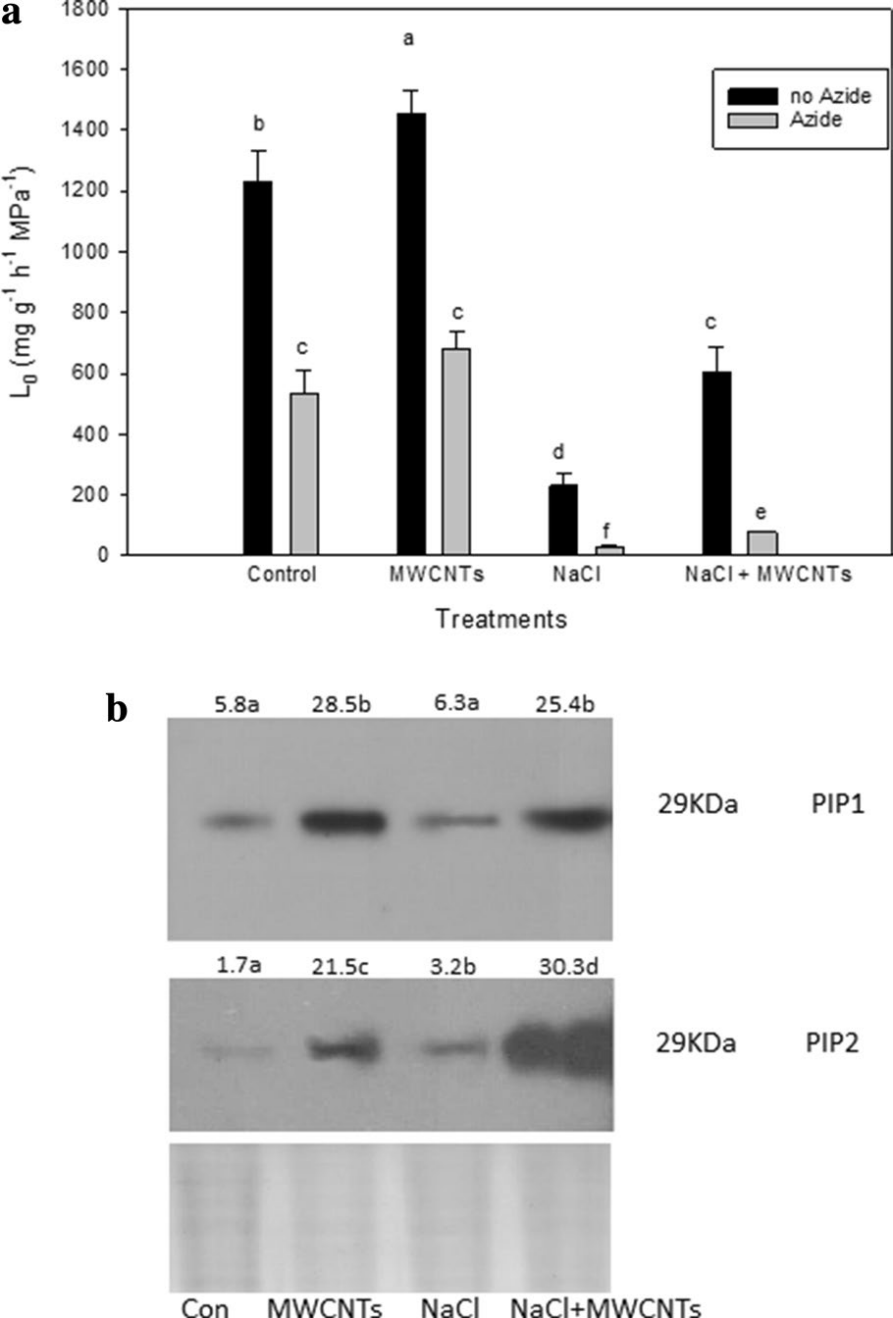
**a** Root hydraulic conductance (L_0_) (mg g^−1^ FW h^−1^ MPa^−1^) of control broccoli plants and plants treated with 10 mg l^−1^ MWCNTs, 100 mM NaCl and 100 mM NaCl + 0^.^10 mg l^−1^ MWCNTs. A group of plants within each treatment was treated with 7 mM sodium azide before measurement of L_0_. Mean values ± standard errors are shown (n = 6). *Bars* with different letters are significantly different (*P* < 0.05) according to the Tukey test. **b** Immunodetection of PIP1 and PIP2 homologues in the root plasma membrane (PM) of control broccoli plantsand plants treated with 10 mg l^−1^ MWCNTs, 100 mM NaCl and 100 mM NaCl + 0^.^10 mg l^−1^ MWCNTs. Total PM was separated by SDS-PAGE and probed with antibody against *At*PIP1;1 and *Bo*PIP2. Equal amounts of protein (10 µg) were loaded in each lane. Mean values are shown (n = 3). *Different letters* represent significant (P < 0.05) differences according to the Tukey test

The root hydraulic conductance (L_0_) of broccoli plants was measured with a Scholander chamber 1 week after applying the different treatments: NaCl, MWCNTs and NaCl + MWCNTs (Fig. 4a). There was an increase in the L_0_ when control plants were treated with MWCNTs. However, L_0_ was significantly lower in plants treated with NaCl and NaCl + MWCNTs, although it was higher in NaCl + MWCNTs plants with respect to NaCl-treated plants.

When plants were treated with sodium azide, L_0_ was lower. Control and MWCNTs-treated plants showed similar reductions of L_0_ (56.47 and 53.26 %, respectively) after sodium azide treatment. The NaCl and NaCl + MWCNTs-treated plants exhibited very-low L_0_ values that were further affected by sodium azide treatment, but again plants exposed to NaCl + MWCNTs showed higher values than NaCl-treated plants.

### Aquaporin protein abundance

For quantification of broccoli PIP1 and PIP2 protein abundance in the roots, Western blotting was performed. A unique band at 29 kDa (monomeric form) was detected with both aquaporin (PIP1 and PIP2) antibodies, in all the samples analysed. No dimeric bands were detected (Fig. 4b). The immunostaining intensity differed among treatments and depended on the PIP subfamily. Thus, similar PIP1 abundance was found in control and NaCl-treated plants, while a significant increase in the PIP1 content due to the MWCNTs treatment was observed under both non-saline and saline conditions. However, PIP2 proteins were increased by both the NaCl and MWCNTs treatments, with respect to the control, but the increment was higher with MWCNTs application. The PIP2 abundance was the highest when the combination of NaCl and MWCNTs was applied.

### Leaf water potential

The leaf water potential (Ψ_ω_) was reduced by salinity (Table 2). There were no significant differences in Ψ_ω_ values between control and MWCNTs-treated plants. However, a Ψ_ω_ restoration was observed in NaCl + MWCNTs-treated plants with regard to those treated with NaCl.

**Table 2:** Leaf water potential (ψ_w_) (MPa) of control broccoli plants and plants treated with 10 mg l^−1^ MWCNTs, 100 mM NaCl and 100 mM NaCl + 0^.^10 mg l^−1^ MWCNTs

	ψ_w (MPa_)
Control	−0.30 ± 0.03a
MWCNTs	−0.25 ± 0.04a
NaCl	−0.73 ± 0.08c
NaCl + MWCNTs	−0.55 ± 0.08b

Mean values ± standard errors are shown (n = 6). Bars with different letters are significantly different (*P* < 0.05) according to the Tukey test

### Effect on the root plasma membrane lipid composition

Table 3 shows the most-significant results: the double bond index (DBI), ratio of unsaturated fatty acids (RUFA), stigmasterol-sitosterol ratio and protein-lipid ratio. Significant differences in the amounts of fatty acids were observed between control and treated plants. In NaCl-treated plants an increase in the DBI was observed and this was greater still with both MWCNTs treatments. However, the RUFA increased significantly in plants treated with MWCNTs or NaCl + MWCNTs but not significantly in NaCl-treated plants.

**Table 3:** Effect of MWCNT on changes in the lipid composition of the plasma membrane of roots of broccoli plants grown for 7 days with distinct treatments

	Control	MWCNTs	NaCl	NaCl + MWCNTs
Fatty acids				
DBI	137.5 ± 5.53 a	151.8 ± 4.14 c	143.6 ± 4.41 b	153.4 ± 2.45 c
RUFA	0.75 ± 0.08 a	1.68 ± 0.01 b	0.95 ± 0.09 a	1.78 ± 0.12 b
Sterols				
Stig/sitos	0.21 ± 0.01 b	0.09 ± 0.01 a	0.63 ± 0.07 b	0.08 ± 0.08 a
Prot/lipid ratio	1.25 ± 0.04 a	1.96 ± 0.09 b	1.38 ± 0.11 a	1.82 ± 0.046 b

Values with different letters are significantly different (n = 6, Tukey, P < 0.05)

*RUFA* ratio of unsaturated fatty acids = (18:2 + 18:3/18:1); *DBI* double bond index = ∑ (unsaturated fatty acids x number of double bonds)

The effect of different treatments on the free sterol concentration of the root plasma membrane was also investigated. The sitosterol concentration was higher in both MWCNTs and NaCl + MWCNTs-treated plants, resulting in very-low stigmasterol/sitosterol ratio. However, NaCl-treated plants showed a higher stigmasterol/sitosterol ratio, due to a decreased sitosterol concentration. The protein/lipid ratio in the root plasma membrane was enhanced by all the treatments, relative to the control, the increase being slightly higher in the two treatments with MWCNTs.

### Subcellular distribution of MWCNTs

**Fig. 5:**
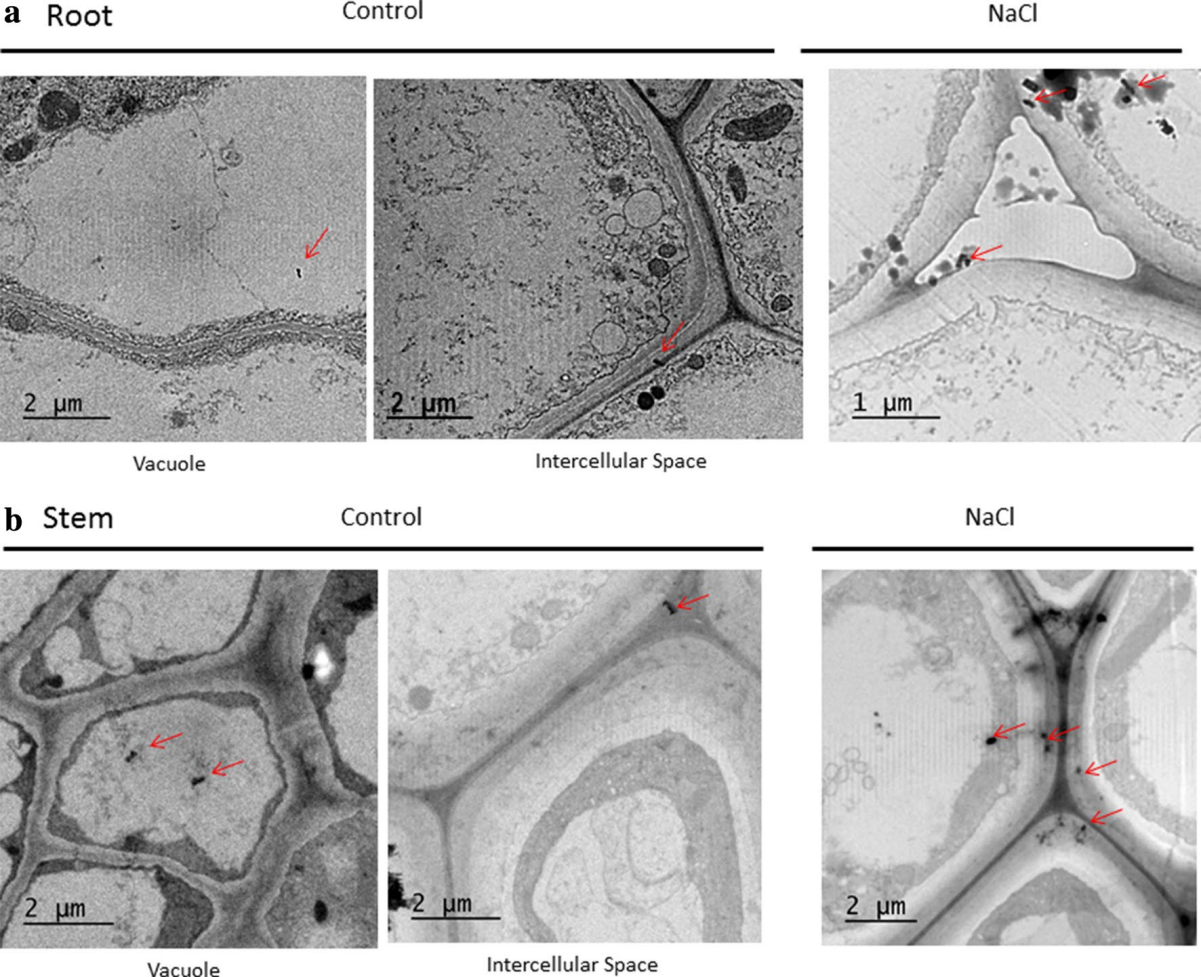
TEM characterization of MWCNTs uptake when adult broccoli plants were grown with MWCNTs (10 mg l^−1^) in the nutrient solution during 7 days. MWCNTs were allocated in different organs, **a** root and **b** stem of control and 100 mM NaCl treated plants. The *arrows* indicate the MWCNTs in intercellular space, vacuole and cytoplasm

Few studies have been determined the subcellular localization of carbon nanotubes in plants. In this work, the direct penetration of MWCNTs into root cells allows MWCNTs could be seen inside of different identified target sites in the root and the stem (Fig. 5). Thus, MWCNTs of different lengths were localized in the cell vacuole, intercellular space and cytoplasm. In leaves, no MWCNTs were detected. In general, NaCl-treated plants showed a higher accumulation of isolated MWCNTs in the vacuole, intercellular spaces and cytoplasm than non-saline plants (Fig. 5).

## Discussion

It has been previously demonstrated that MWCNTs can penetrate seed coats and promote germination and growth [9, 48, 49]. It has been observed that CNTs may promote water uptake as a growth stimulator [12, 50] with an increased expression of *PIP1* genes in “in vitro*”* tobacco cell cultures [32]. However, the effect of MWCNTs on the plasma membrane properties and its ability to transport water under saline- stress conditions has not been studied. Controversial results of beneficial or toxic implications of MWCNTs on plant growth has been reported [51], and different observations depended mainly on the plant genotype, plant growth medium, environmental conditions, type of MWCNTs, their size, concentration and chemical surface functionalization [52].

Our transmission electron microscopy (TEM) analysis determined that 75 % of the nanotubes had 0.1–0.5 μm length in spite those manufacturer specifications (0.5–10 μm length). This size after ultrasonication to disperse MWCNTs in the aqueous solution and the high-purity of MWCNTs used (95 %) had a positive effect on the growth of broccoli plants at the seedling stage under both, saline and non-saline conditions.

The application of the treatments was performed in adult plants when they were assumed to have a mineral-nutrient status consistent with optimum growth and the plants were grown in a hydroponic medium to allow the MWCNTs to enter the roots by more-direct penetration.

Studies of transport and translocation of nanotubes assume that there is no interaction with the substances present on the root surface in order to allow CNTs adsorption [20]. Thus, penetration could be taken place through the apoplastic pathway by capillarity until arrive at a narrower passage than their size, being then accumulated with impediment of nutrient transport. However, in our broccoli plants the MWCNTs diameter and length and their concentration in the media were not limiting factors for the nutrient uptake availability of the plant. As previously done by Begun and Fugetsu [51] for spinach, the MWCNTs were applied to a Hoagland nutrient solution, but at lower concentrations. These authors applied a range of MWCNTs concentrations from 125 to 1000 mg l^−1^, with no positive effects on plant growth. For our broccoli plants an interval from 10 to 60 mg l^−1^ was used: 10 mg l^−1^ MWCNTs gave a significant increase of growth but the rest of the concentrations produced perturbations. Improved growth due to MWCNTs application was related to increases in the nutrient and water uptake in maize cell cultured plants [19]. The authors considered the action of opposing forces, the CNTs ion-transient-dipole interaction, that retain ions in the roots and promote the water flow that facilitates further ion uptake. Therefore, an optimum concentration of MWCNTs was related to the effect of these opposing forces. In our broccoli roots, MWCNTs originated an increase in the Na concentration relative to NaCl-treated plants, but the rest of tissue nutrient concentrations were not altered by any of the treatments. Our results are in agreement with those reported by Husen and Siddiqi [20], where essential plant nutrients were absorbed by the plant in the presence of nanomaterials suspended in aqueous media. However, variations between our results and those of Tiwari et al. [19], who observed differences for most of nutrients, could be related to the type of growth medium—nutrient agar gel vs. nutrient solution, the aqueous dispersion of the MWCNTs being greater in the latter—and to the experimental entity, maize seedlings vs. adult broccoli plants, affecting the root content of the most abundant cation, Na, in the nutrient solution. Root Na rise could be also the consequence of increased water uptake that was more efficient in MWCNTs-treated plants, which may be related directly to their greater growth.

Broccoli plants are moderately salt tolerant and, therefore, can cope with low external salinity, with only a slight decrease in growth [47]. In our work, the NaCl concentration (100 mM) was higher than the maximum assayed in previous studies (80 mM), being based on the level at which growth decreased during a limited experimental exposure time. Thus, in previous report, we observed a biphasic growth inhibition of broccoli plants in response to salinity and during the second phase the internal injury was due to accumulation of salts in transpiring leaves [47]. In this work, similar K/Na ratios were observed in the shoots of NaCl and NaCl + MWCNTs-treated plants. Thus, the maintenance of a high K/Na ratio for salt tolerance was not the operative mechanism of MWCNTs.

Salinity in broccoli plants was also related to alterations of the membrane lipid composition and fluidity—that contribute to reductions of cell expansion [31]. This response was related to water uptake and transport—regulated by aquaporins functionality and abundance, key factors in the movement of water into the plant. The present work investigated whether or not the MWCNTs interfere with the response of broccoli plants to salinity, since they have been reported to target membranes and aquaporins. The application of MWCNTs increased plant growth, the increment being higher in NaCl-treated plants, and the amelioration of the salinity effect was very apparent after 7 days of MWCNTs treatment. In control and NaCl-treated plants supplied with MWCNTs, the greater presence of PIP1 and PIP2 aquaporins corroborated the increase in water passage through the plasma membrane that was related to higher L_0_. The stimulation of aquaporins production in in vitro tomato plants by MWCNT has been reported [48] as the main factor inducing growth, but other genes involved in cell division have been activated also [10] and cannot be ruled out in our broccoli plants.

It has been widely reported that salinity decreases stomatal conductance (G_s_) and CO_2_ fixation [27]. In broccoli plants, these results have been related to both stomatal aperture and photosynthetic rate or net CO_2_ assimilation (ACO_2_) [53]; stomatal closure resulted in a decrease in leaf transpiration and leaf internal CO_2_ concentration. Also, it has been reported that plant cell homeostasis in NaCl-treated plants must involve the combined regulation of L_0_ and G_s_, with a consequent dependence on root aquaporins [54]. In our plants, MWCNTs slightly increased G_s_ and ACO_2_, even in the NaCl treatment, and a correlation between stomatal behaviour and L_0_ was observed under non-saline and saline conditions. Therefore, the simultaneous rises in G_s_ and L_0_ induced by MWCNTs may indicate enhanced symplastic movement of water, through an increased abundance of aquaporins, which maintained the cellular water balance, ψ_w_ and, in turn, augmented plant growth. Under saline conditions, the increment in L_0_ and ψ_w_ could have been related, in addition to the greater aquaporin abundance, to higher aquaporin functionality, as deduced from the sodium azide experiments. In previous work, HgCl_2_, a mercurial inhibitor of aquaporin functionality, was used; the reversal of its effect by DTT was used to demonstrate the absence of side effects [55]. Also, K^+^ leakage was used as a control [29]. Mercurials act through covalent union with cysteine residues of aquaporin proteins, within the water pore and in other regions of the protein, causing either blockage or conformational changes that lead to inhibition of water transport [56]. Thus, the use of alternative compounds such as azide, that inhibits protein phosphorylation and causes acidification of the cytoplasm, hence contributing to closure of the aquaporin channel [40], has been very useful due to the absence of side effects [41]. The azide results may demonstrate the involvement of the symplastic route of water movement in the NaCl + MWCNTs-treated plants with higher L_0_ reductions than control plants. But the results must be carefully interpreted since mutagenic effect of sodium azide on growth and yield traits of *Eruca sativa* (L.), another Brassicaceae, has been observed when the compound was applied at a concentration of 3 mM [57].

Carbon nanotubes can be taken up by roots and moved to the leaves [20]. Although interaction with mineral nutrients has not been claimed to be the driving force for apoplastic transport of MWCNTs, the driving force of water should allow their movement through the plant [20]. Also, their penetration through the cell wall and membranes has been observed only on some occasions, when the concentration of the nanotubes applied was high (100–1000 mg l^−1^) [58]. However, this not the case of our broccoli plants and MWCNTs pass through the cell wall in the plant mature stage and they were observed in intercellular spaces, vacuole, plasma membrane and cytoplasm in the cortical cells of the roots and stem in control and NaCl-treated plants. This could be related to the size and low concentration used (10 mg l^−1^) to the rate of movement through the symplastic/apoplastic pathway—that allowed MWCNT passively diffused across the cells. Also MWCNTs distribution in broccoli plants organelles was influenced by their size, high resolution transmission electron microscopy showed that long MWCNTs (larger than 200 nm) were concentrated in subcellular organelles while the shorter ones (30–100 nm) were acquired into vacuoles, nucleus and plastids [59, 60]. Similarly, Chen et al. [61] demonstrated that the MWCNTs could be translocated from the roots to the upper organs within mustard mature plants under the transpiration stream. However, in our broccoli plants MWCNTs were not found in the leaves of any plants. A diffusion and leakage of MWCNTs in the aerial part must be not discarded as it was observed for SWCNTs when the size of particle is too small [59, 62]. By contrast, higher accumulations were observed in NaCl + MWCNTs-treated plants with lower G_s_ values that MWCNTs-treated plants. Thus, MWCNTs accumulation should be investigated in terms of the action driven by the root water pressure.

Also, changes in the plasma membrane composition could be very important in relation to MWCNTs penetration within our broccoli plants. Although it has been pointed out that CNTs penetrate cells directly rather than through endocytosis [63] and that they can act as molecular transporters in walled cells [59], their passage through membranes seems to be related to the membrane stability and to lipid trapping by lipid exchange [64]. Therefore, the changes in control lipid composition in our membranes were a response to the MWCNTs and salinity. The sitosterol/stigmasterol ratio was increased in the plasma membranes of plants exposed to salinity, indicating a swift increase in water permeability, since sitosterol has been found to be very efficient in regulating water permeability and salt protection [65, 66]. However, the ratio was strongly decreased by the MWCNTs, which have been reported to give rise to more-stable membranes in the raft domains [67]. This effect should be investigated; it could form part of a mechanism to compensate for greater water movement through the increased number of aquaporin proteins.

The increase in the DBI and RUFA of the fatty acid content in the leaf plasma membrane of NaCl-treated plants has been reported to be related to reductions in membrane fluidity [68]. But, these changes reduce fluidity, increasing water permeability [65]. The increments in DBI and RUFA in our root plasma membrane as a consequence of the application of MWCNTs were higher than those induced by salinity; therefore, these carbon compounds produced more rigidity but probably also increased membrane permeability, relative to salt stress.

Recently, it has been shown that plasma membrane lipid modifications in broccoli plants in response to salinity occurred in order to maintain protein function and ensure membrane permeability [36]. Also, the increased content of proteins, such as aquaporins, is relevant with regard to promotion of ion and water transport and maintenance of the electrostatic gradient across the plasma membrane, and hence cell homeostasis, but also for decreasing damage to the membrane and ensuring its stability [37]. Nevertheless, the observation that biological membranes are able to form ordered lipid domains (raft domains) that are highly stable [69] and enriched in aquaporins [70], but with unknown function, reinforces the idea that the variability of plasma membrane composition and fluidity, as a response to salinity and MWCNTs, may be the key to understanding their physiological effects.

## Conclusions

The results presented in this work reinforce and build upon previous results obtained with the application of CNTs to plants cultured in vitro, extending the approach to adult plants. Also, the novel use of salt-stressed plants has shown that the MWCNTs influenced the primary plant response in relation to water and nutrient uptake. The positive biochemical changes found could simply have been due to the known physical changes produced in cells and tissues by the MWCNTs and under salt stress these compounds may retain Na ions in the root that promote water uptake. The plants appeared able to cope with changes in the water gradient through the imposed symplastic pathway, giving positive results. But, these events are only the starting point of a chain of biochemical changes that should be investigated as well as the movement of MWCNTs to edible part of the plants (e.g: leaves and fruits) and the environmental implications. In conclusion, the positive growth response by the broccoli plants to low concentrations of MWCNTs opens up new perspectives for the use of MWCNTs in agriculture, especially when plants are growing under stress.

## Additional files

10.1186/s12951-016-0199-4 Effect of different MWCNTs concentrations on Fresh weight (FW) (g plant^−1^). Mean values ± standard errors are shown (n = 6). Bars with different letters are significantly different (*P* < 0.05) according to the Tukey test.
10.1186/s12951-016-0199-4 Fresh weight (FW) (g plant−^1^) of control broccoli plants and plants treated with 80, 100, and 120 mM NaCl. Mean values ± standard errors are shown (n = 6). Bars with different letters are significantly different (*P* < 0.05) according to the Tukey test.
10.1186/s12951-016-0199-4 Dry weight (DW) (g plant−^1^) of control broccoli plants and plants treated with 80, 100, and 120 mM NaCl. Mean values ± standard errors are shown (n = 6). Bars with different letters are significantly different (*P* < 0.05) according to the Tukey test.
